# The Dual Role of Exogenous Hydrogen Sulfide (H_2_S) in Intestinal Barrier Mitochondrial Function: Insights into Cytoprotection and Cytotoxicity Under Non-Stressed Conditions

**DOI:** 10.3390/antiox14040384

**Published:** 2025-03-25

**Authors:** Domenica Mallardi, Guglielmina Chimienti, Fatima Maqoud, Antonella Orlando, Simona Drago, Eleonora Malerba, Caterina De Virgilio, Hamid I. Akbarali, Francesco Russo

**Affiliations:** 1 Functional Gastrointestinal Disorders Research Group, National Institute of Gastroenterology IRCCS “Saverio de Bellis”, Castellana Grotte, 70013 Bari, Italy; domenica.mallardi@irccsdebellis.it (D.M.); fatima.maqoud@irccsdebellis.it (F.M.); antonella.orlando@irccsdebellis.it (A.O.); simona.drago@irccsdebellis.it (S.D.); eleonora.malerba@irccsdebellis.it (E.M.); 2 Department of Biosciences, Biotechnologies and Environment, University of Bari Aldo Moro, 70125 Bari, Italy; guglielminaalessandra.chimienti@uniba.it (G.C.); caterina.devirgilio@uniba.it (C.D.V.); 3 Department of Pharmacology and Toxicology, School of Medicine, Virginia Commonwealth University, Richmond, VA 23298, USA; hamid.akbarali@vcuhealth.org

**Keywords:** Caco-2 monolayer model, hydrogen sulfide, cytoprotection, oxidation reduction, mitochondria/metabolism, gastrointestinal tract

## Abstract

Hydrogen sulfide (H_2_S) is a critical gasotransmitter that plays a dual role in physiological and pathological processes, particularly in the gastrointestinal tract. While physiological levels of H_2_S exert cytoprotective effects, excessive concentrations can lead to toxicity, oxidative stress, and inflammation. The aim of this study was to investigate the dose-dependent effects of exogenous H_2_S on mitochondrial functions and biogenesis in intestinal epithelial cells under non-stressed conditions. Using a Caco-2 monolayer model, we evaluated the impact of sodium hydrosulfide (NaHS) at concentrations ranging from 1 × 10^−7^ M to 5 × 10^−3^ M on mitochondrial metabolism, redox balance, antioxidant defense, inflammatory responses, autophagy/mitophagy, and apoptosis. Our results demonstrated a biphasic response: low-to-moderate H_2_S concentrations (1 × 10^−7^ M–1.5 × 10^−3^ M) enhance mitochondrial biogenesis through PGC-1α activation, upregulating TFAM and COX-4 expression, and increasing the mtDNA copy number. In contrast, higher concentrations (2 × 10^−3^–5 × 10^−3^ M) impair mitochondrial function, induce oxidative stress, and promote apoptosis. These effects are associated with elevated reactive oxygen species (ROS) production, dysregulation of antioxidant enzymes, and COX-2-mediated inflammation. H_2_S-induced autophagy/mitophagy is a protective mechanism at intermediate concentrations but fails to mitigate mitochondrial damage at toxic levels. This study underscores the delicate balance between the cytoprotective and cytotoxic effects of exogenous H_2_S in intestinal cells, helping to develop new therapeutic approaches for gastrointestinal disorders.

## 1. Introduction

Hydrogen sulfide (H_2_S) is the third gaseous signaling molecule, alongside nitric oxide (NO) and carbon monoxide (CO). As a versatile gasotransmitter, H_2_S plays critical roles in various biological systems, including the gastrointestinal (GI), nervous, cardiovascular, respiratory, renal, and hepatic systems, exhibiting physiological and pathological functions. While high levels of H_2_S are toxic and pose significant health risks, its therapeutic potential has garnered increasing attention, particularly for addressing inflammation, visceral pain, oxidative stress, thrombosis, and cancer [1]. Notably, H_2_S exerts a dual role in mitochondrial metabolism: it can stimulate mitochondrial electron transport by acting as an electron donor to complex II of the electron transport chain (ETC) [2], but it can also inhibit complex IV, disrupting ETC activity and energy production [3].

In the GI tract, H_2_S is produced endogenously by epithelial cells through cysteine metabolism and exogenously by gut microbiota. Endogenous production is mediated by key enzymes, including cystathionine β-synthase (CBS), cystathionine γ-lyase (CSE), and 3-mercaptopyruvate sulfurtransferase (3MST), with CBS being the primary enzyme in colonocytes. On the other hand, gut microbiota serves as a significant source of H_2_S, primarily through the breakdown of cysteine by bacteria such as Fusobacterium, Clostridium, Escherichia, Salmonella, Klebsiella, Streptococcus, Desulfovibrio, and Enterobacter. These bacteria utilize cysteine desulfhydrase to convert cysteine into H_2_S, pyruvate, and ammonia. Additionally, sulfate-reducing bacteria (SRB) contribute to H_2_S production, albeit to a lesser extent [4].

H_2_S regulates intestinal processes such as inflammation, ischemia/reperfusion injury, and motility. However, in dysbiosis, excessive H_2_S production by gut microbiota is linked to GI disorders, including ulcerative colitis, Crohn’s disease, and irritable bowel syndrome (IBS). Beyond its pathological implications, H_2_S is essential for maintaining intestinal physiology, influencing blood flow, motility, and epithelial barrier permeability [5]. Notably, the dysregulated intestinal barrier, which acts as a barrier between the mucosal immune system and the external environment, has a role in inflammatory diseases inside and outside the GI tract [6]. Recent studies have explored the therapeutic potential of H_2_S donors in reducing colonic inflammation, restoring microbiota balance, and regulating mucosal homeostasis, as demonstrated in animal models of colitis [7].

A key cellular mechanism for managing energy demands, whether physiological or pathological, is the regulation of mitochondrial biogenesis. This process involves the growth and division of existing mitochondria and the selective removal of damaged organelles through mitochondrial autophagy. Significant attention has been given to the signaling pathways that activate mitochondrial biogenesis during oxidative stress [8]. Similar to other gasotransmitters [9,10], endogenous H_2_S plays a crucial role in regulating oxidative metabolism to maintain energy homeostasis, primarily through the modulation of the mitochondrial transcriptional coactivator Peroxisome proliferator-activated receptor-γ coactivator (PGC)-1α [11,12]. PGC-1α is the master regulator of mitochondrial biogenesis and function, including detoxification of reactive oxygen species (ROS) [13]. It serves as a critical link between mitochondrial metabolism, oxidative stress, and inflammatory responses, regulating the expression of mitochondrial antioxidant genes. Dysregulation of PGC-1α disrupts redox homeostasis, exacerbating inflammatory processes [14].

Emerging evidence underscores the central role of H_2_S in mitigating oxidative stress. H_2_S modulates the activity and expression levels of antioxidant enzymes and achieves this through persulfidation, a post-translational modification of key proteins that influences their function [15,16]. This modification enables H_2_S to regulate the cellular signaling pathways involved in autophagy, inflammation, and stress responses, further highlighting its multifaceted role in cellular physiology [17]. Moreover, H_2_S is among the few molecules capable of inducing mitochondrial biogenesis without prior organelle damage, underscoring its unique therapeutic potential.

Based on these premises, the present study aimed to investigate the dual role of H_2_S in the gut, focusing on the balance between its protective and toxic effects and its therapeutic potential in GI diseases. Specifically, since the significant role of mitochondrial dysfunction in disorders such as IBD, IBS, and celiac disease has been demonstrated [18,19,20], we explored how H_2_S regulates mitochondrial metabolism and biogenesis to maintain redox homeostasis and mitigate inflammation,

Using a Caco-2 monolayer model, we treated these intestinal cells with exogenous H_2_S (as sodium hydrosulfide, NaHS, at 1 × 10^−7^ M^−5^ × 10^−3^ M) under non-stressed conditions to identify the concentration threshold at which H_2_S transitions from cytoprotective to cytotoxic and to elucidate the underlying molecular mechanisms. Key analyses included mitochondrial metabolic activity, cellular viability, ROS levels, and markers of mitochondrial biogenesis (PGC-1α, TFAM, COX-4, and mtDNA copy number). We also assessed the antioxidant responses (SOD1/2), redox status (oxidatively modified mtDNA purines), inflammation (COX-2), and autophagy/mitophagy markers (Beclin-1, LC3). The possible induction of an apoptotic response was also evaluated (Bax/BCL-2 ratio). Untreated cells served as controls.

The obtained findings could advance the understanding of the role of H_2_S in mitochondrial biogenesis and redox balance, offering new therapeutic strategies for GI diseases.

## 2. Materials and Methods

### 2.1. Cell Culture and Treatments

A human colon adenocarcinoma-derived Caco-2 cell line was cultured in DMEM with 10% FBS, 1% penicillin–streptomycin, and 1% MEM at 37 °C and 5% CO_2_, changing the medium every 2–3 days until they formed a monolayer culture, 80–90% confluent. For these experiments, sodium hydrosulfide (NaHS) was used as a fast H_2_S-releasing donor (Sigma-Aldrich, Burlington, MA, USA). Cells were used in passages 10–15 to ensure a low variability in our results. At confluence, the cells were treated in apical compartments with different concentrations (from 1 × 10^−7^ M to 5 × 10^−3^ M) of NaHS for 24 h. Each treatment included its control (untreated cells).

### 2.2. Metabolic Viability Assay (MTT)

The metabolic viability of H_2_S in Caco-2 cells was assessed using the MTT assay (3-(4,5-dimethylthiazol-2yl)-2,5-diphenyl-tetrazolium bromide). This method measures the reduction of the tetrazolium salt MTT into insoluble purple formazan crystals by mitochondrial dehydrogenases, predominantly succinate dehydrogenase, in metabolically active cells [21].

Briefly, Caco-2 cells were seeded at a density of 4 × 10^5^ cells per well in 24-well culture plates and incubated overnight in a CO_2_ incubator. The following day, treatments with NaHS were administered at concentrations ranging from 1 × 10^−7^ M to 5 × 10^−3^ M, and the cells were incubated for 24 h. After incubation, 50 µL of the MTT solution (5 mg/mL) was added to each well containing 1 mL of DMEM medium, and the plates were incubated for an additional 3 h at 37 °C in the dark. Subsequently, the medium was carefully removed, and 150 µL of DMSO was added to each well to dissolve the formazan crystals. The absorbance of the solubilized formazan was measured at 570 nm using a UV spectrophotometer. The quantity of formazan formed was directly proportional to the number of viable, metabolically active cells, indicating cellular viability.

### 2.3. Crystal Violet Cell Proliferation Assay

This test is based on the logic that cells in the cell death process lose their adhesion and detach from the cell culture plate, thus reducing the amount of staining dye. Based on these properties, Crystal Violet staining directly relates to the attached cell biomass (indicative of viable cells). Briefly, Caco-2 cells were seeded at a density of 4 × 10^4^ cells per well in 24-well culture plates and incubated overnight in a CO_2_ incubator. The following day, treatments with H_2_S were administered at concentrations ranging from 1 × 10^−7^ M to 5 × 10^−3^ M, and the cells were incubated for 24 h. At the end of the treatment, the cells were fixed for 10 min in a buffered formalin solution (3.7%), washed with PBS (pH 7.3), and subsequently stained with a 0.01% crystal violet solution in PBS. After carefully removing the excess dye, the crystal violet-stained cells were dissolved in 1 mL of methanol, and the optical density of the extracted dye was read with a spectrophotometer at 590 nm.

### 2.4. Reactive Oxygen Species (ROS) Production Assessment

ROS production after NaHS treatment was monitored using DCFH-DA. Briefly, Caco-2 cells were plated at 1 × 10^4^ cells in dark 96-well plates with clear bottoms. After 24 h of stabilization, the growth medium was replaced with 300 µL of clear DMEM without FBS containing 4 µM DCFH-DA for 30 min, followed by an increasing dose treatment with NaHS. After incubation for another 30 min, the cells were washed, and the fluorescence intensity of the resulting DCF was quantitatively measured using a microplate reader with 485 nm excitation and 535 nm emission filters. Data are presented as percentage values compared to the control.

### 2.5. Western Blotting Analysis

The untreated controls and treated cells were pelleted, and protein was extracted with lysis buffer (Pierce RIPA buffer, manufactured by Thermo Scientific (Rockford, IL, USA)) and protease and phosphatase inhibitors (Thermo Scientific, Rockford, IL, USA). After homogenization and centrifugation at 14,000 rpm for 20 min at 4 °C, the protein concentration was determined using a standard Bradford assay (Bio-Rad, Milan, Italy). Subsequently, 80 µg aliquots of total protein extracts from each sample were denatured in a 4× Laemmli sample buffer at 70 °C for 10 min and loaded into pre-cast polyacrylamide gels (4–12%) from Bio-Rad (Milan, Italy) for Western blot analysis. The samples were subjected to SDS-PAGE electrophoresis and membrane transfer, sealed with 5% milk solution, and cut into corresponding bands. The membranes were washed four times for 5 min each with TBS solution and supplemented with 0.1% Tween-20 (TBST). The membranes were washed with TBST and incubated overnight at 4 °C with a dilution of primary antibodies: superoxide dismutase 1 (SOD1) (Cell Signaling Technology, Danvers, MA, USA, 37385), superoxide dismutase 2 (SOD2) (Cell Signaling Technology, Danvers, MA, USA, 13141), TFAM (Cell Signaling, Danvers, MA, USA, 7495), PGC-1α, Beclin-1 (Cell Signaling Technology, Danvers, MA, USA, 3738), COX-2, COX-4 (Cell Signaling Technology, Danvers, MA, USA, 12282), and-β-actin (Cell Signaling Technology, Danvers, MA, USA, 4970).

Then, the bands were rewashed with TBST and incubated for 1 h at room temperature with secondary antibodies: Anti-Rabbit IgG (1:10,000 dilutions, 211-032-171; Jackson ImmunoResearch Inc., West Grove, PA, USA) or Anti-mouse IgG, HRP-linked Antibody (1:1000 dilution, 7076; Cell Signaling Technology, Danvers, MA, USA) for 1 h at room temperature (20–25 °C). The blots were washed four times for 5 min each with PBST.

The proteins were detected by Chemiluminescence (Clarity Western ECL substrate, Bio-Rad, Milan, Italy), analyzing signals with the ChemiDoc System (Model No. Universal Hood III), and Image Lab version 6.1 software from Bio-Rad Laboratories Inc. (Hercules, CA, USA). To normalize each band’s densitometric values (OD units), the β-actin expression was utilized as a reference.

### 2.6. Assessment of Mitochondrial DNA Copy Number

Total genomic DNA was isolated from the Caco-2 cells (2 × 10^6^) cultured in the different experimental conditions using the DNeasy Blood and Tissue Kit (QIAGEN, Hilden, Germany), according to the manufacturer’s instructions. The copy number of the mitochondrial DNA relative to the GAPDH nuclear gene was determined using a quantitative real-time polymerase chain reaction (qRT-PCR) via SYBR Green chemistry. Reactions were performed in triplicate with a final volume of 10 μL containing 10 ng of total DNA, 0.2 μM forward and reverse primers, and 1 × iTaq Universal SYBR Green Supermix (Bio-Rad Laboratories Inc., Hercules, CA, USA) [22]. The PCR conditions were as follows: initial denaturation at 95 °C for 10 min, followed by 40 cycles at 95 °C for 5 s, and annealing and extension at 60 °C for 30 s. The relative mtDNA content (Ct ND1-Ct GAPDH) was determined using the 2^−ΔCt^ method [23]. The primer sequences are reported in Table 1.

### 2.7. Quantification of mtDNA Oxidized Purines

The abundance of oxidized purines in the D-Loop region of the mtDNA was evaluated using formamidopyrimidine DNA glycosylase (Fpg) (New England Biolabs, Beverly, MA, USA) digestion of total DNA. The method is based on the selective block of amplification due to the single-strand breaks introduced by Fpg at the sites of oxidized purines. The PCR amplification was conducted on Fpg-treated and untreated samples using an RT-PCR-based SYBR Green chemistry detection method, as in Saini et al. [22]. Briefly, 250 ng of the total DNA was incubated with 8 U of Fpg at 37 °C for 1 h. Untreated DNA served as the negative control. The reaction was stopped by heating at 60 °C for 5 min. RT-PCR reactions were performed in triplicate in a final volume of 20 µL containing 15 ng of the template DNA, 0.5 μM forward and reverse D-loop primers, and 1 ×iTaq Universal SYBR Green Supermix (Bio-Rad Laboratories Inc., Hercules, CA, USA). The relative abundance of oxidative damage was calculated using the formula R = 2^(Ct Fpg-treated − Ct untreated negative control)^. The primer sequences are reported in Table 1.

### 2.8. Immunofluorescence Staining

Caco-2 cells (1 × 10^5^ cells/well) were seeded in 24-well transwell plates and cultured for 15 days to establish a polarized epithelial monolayer. The cells were then exposed to increasing concentrations of NaHS for 24 h, washed with PBS, fixed in 4% paraformaldehyde at 4 °C for 20 min, and permeabilized with 0.5% Triton X-100. After blocking with 1% normal serum in PBS for 1 h, the cells were incubated overnight at 4 °C with an anti-LC3A/B primary antibody (1:200, Cell Signaling). This was followed by a 1 h incubation with Alexa 488-conjugated secondary antibody (1:1000, Invitrogen, Waltham, MA, USA) and mounting with Prolong Gold Antifade containing DAPI. Fluorescence imaging was performed using a Nikon Eclipse Ti2 microscope and analyzed with ImageJ1.54p (National Institutes of Health).

### 2.9. Statistical Analysis

Data are presented as the mean ± standard error of the mean (SEM) or standard deviation (SD) from at least three independent experiments, as specified. Normality was assessed using the Shapiro–Wilk test. A one-way analysis of variance (ANOVA) was performed for comparisons between multiple groups, followed by Dunnett’s post hoc test to compare the treated groups to the untreated control. A *p*-value of < 0.05 (* *p* < 0.05, ** *p* < 0.01, ***^,^ **** *p* < 0.001). was considered statistically significant.

Linear regression analysis was conducted to evaluate the correlations between variables, with the Pearson correlation coefficients (R) and coefficients of determination (R^2^) reported to quantify the strength and direction of the relationships. For the biphasic responses, the dose-dependent trends were analyzed using nonlinear regression models. All experiments included the appropriate controls, and technical replicates were averaged for each biological replicate. Statistical significance is indicated in the figures as follows: * *p* < 0.05, ** *p* < 0.01, and *** *p* < 0.001.

## 3. Results

### 3.1. Effects of Treatment with Different Concentrations of H_2_S-Releasing Donor on Mitochondrial Metabolic Activity and Viability in Caco-2 Cells

The impact of NaHS on mitochondrial metabolism in the Caco-2 monolayer model was assessed using the MTT assay, which measures mitochondrial dehydrogenase activity. The cells were treated with increasing concentrations of NaHS to evaluate its effects on metabolic activity.

**Figure 1:**
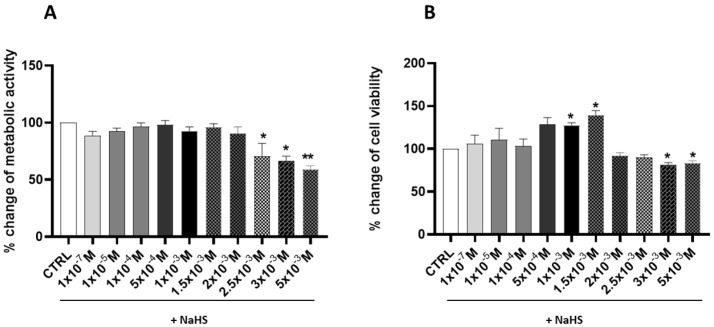
Effects of different concentrations of NaHS on metabolic activity and cell viability of Caco-2 cells after 24 h of treatment. (**A**) The percentage change in the metabolic activity of the cells was measured using the MTT assay. (**B**) The percentage change of the cell viability was measured using the crystal violet assay. The results are expressed as the mean ± SEM of at least three independent experiments. * *p* < 0.05, ** *p* < 0.01 vs. the control group (CTRL).

Following a 24 h treatment, a significant (*p* < 0.05) biphasic biological response was observed (Figure 1A).

The highest concentrations of NaHS effectively impaired mitochondrial metabolic activity, as demonstrated by a significant reduction compared to the untreated control (CTRL) cells (showed by the cells treated with 2.5 × 10^−3^ M, 3 × 10^−3^ M, and 5 × 10^−3^ M); on the contrary, at the lower concentrations, the mitochondrial dehydrogenase activity remains, not showing any significant change. Furthermore, the percentage changes in cell viability, as determined by the crystal violet assay (Figure 1B), demonstrated that NaHS concentrations of 1 × 10^−3^ M and 1.5 × 10^−3^ M significantly increased cell viability by 27.76% and 39.73%, respectively (*p* < 0.05).

In contrast, cell viability was significantly reduced at higher concentrations, with decreases of 18.15% and 25.23% observed at NaHS concentrations of 3 × 10^−3^ M and 5 × 10^−3^ M, respectively (*p* < 0.05). These initial findings underscore the biphasic effect of NaHS on cell viability. In the range of 1 × 10^−7^ M to 1.5 × 10^−3^ M, an increasing trend in cell viability was observed, accompanied by stable metabolic activity. Conversely, at concentrations from 2 × 10^−3^ M to 5 × 10^−3^ M, this effect was reversed, marked by a significant reduction in cell viability and metabolic activity.

### 3.2. Effects of Treatment with Different Concentrations of H_2_S-Releasing Donor on Oxidative Stress

The investigation of ROS production following treatment with the H_2_S donor NaHS revealed a nuanced, dose-dependent response. Fluorescence intensity measurements, used as a proxy for the ROS levels, indicated a non-significant increase at lower NaHS concentrations compared to the CTRL cells. This modest elevation showed variability, particularly at intermediate doses.

**Figure 2:**
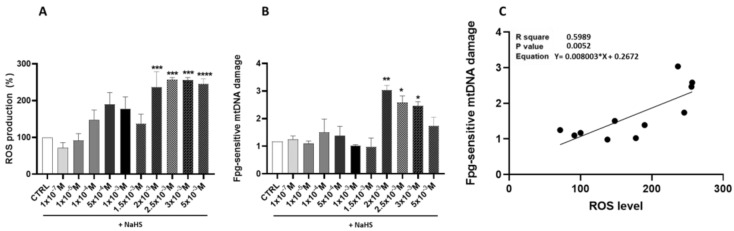
Impact of NaHS treatment on ROS production, mtDNA oxidative damage, and their correlation: (**A**) % change of the DCF fluorescence intensity, indicating ROS production in response to the NaHS treatment in Caco-2 Cells. (**B**) Effect of the NaHS treatment at incremental scaling concentrations on oxidative damage to mtDNA. Oxidative damage was evaluated by quantifying the levels of oxidized purines in the D-loop region of mtDNA using RT-PCR. (**C**) Linear regression showing the correlation between Fpg-sensitive mtDNA damage and ROS levels. Data are presented as the mean ± SD from at least three independent experiments. Statistical significance relative to the untreated control cells is indicated (* *p* < 0.05, ** *p* < 0.01, ***^,^ **** *p* < 0.001).

This modest elevation exhibited variability, especially at intermediate doses. In contrast, NaHS concentrations starting at 2 × 10^−3^ M elicited a marked and statistically significant increase in fluorescence intensity (*p* < 0.05), indicative of elevated ROS production and substantial oxidative stress. These findings suggest the presence of a critical threshold beyond which NaHS exposure disrupts cellular redox equilibrium (Figure 2A).

The potential effect of NaHS supplementation on mtDNA oxidative damage was evaluated. A biphasic pattern of oxidative damage emerged, with statistically significant increases observed at NaHS concentrations of 2 × 10^−3^ M, 2.5 × 10^−3^ M, and 3 × 10^−3^ M compared to the CTRL cells (*p* < 0.05) (Figure 2B). A statistically significant positive correlation was found between the Fpg-sensitive mtDNA damage and ROS levels (R^2^ = 0.60, *p* = 0.0052). These data suggest that excessive levels of ROS produced at high NaHS concentrations can damage mtDNA, ultimately disrupting mitochondrial function and triggering a self-perpetuating cycle of amplified ROS production (Figure 2C).

### 3.3. Effects of Treatment with Different Concentrations of H_2_S-Releasing Donor on Mitochondrial Biogenesis

The potential effects of NaHS supplementation on mitochondrial biogenesis were evaluated by assessing the relative levels of the transcription coactivator PGC-1α, its downstream regulated protein, the mitochondrial transcription factor TFAM, the mitochondrial mass marker cytochrome c oxidase subunit 4 (COX-4), and the relative mtDNA copy number. 

**Figure 3:**
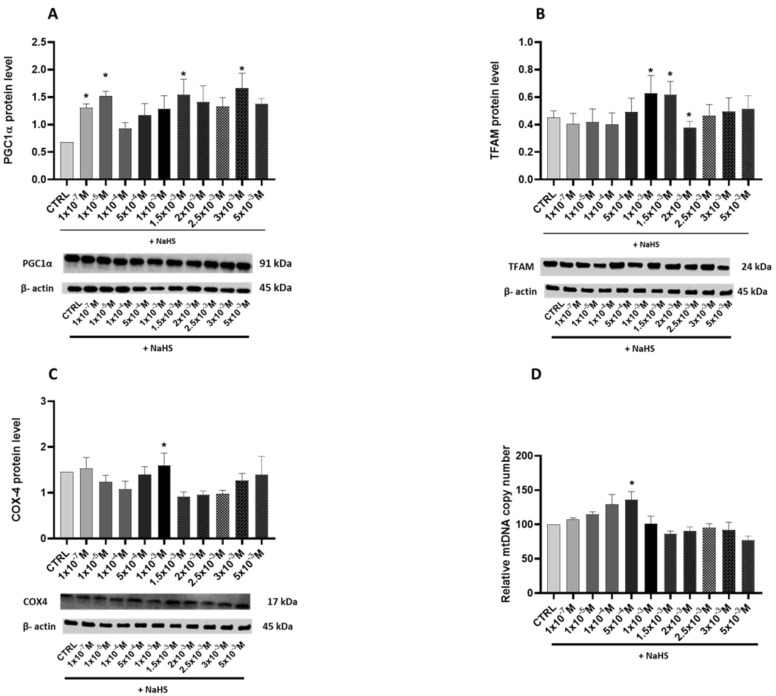
Relative expression levels of the key proteins involved in mitochondrial biogenesis and the mtDNA relative copy number in the Caco-2 monolayer model treated with increasing concentrations of NaHS. Protein abundance was assessed by Western blotting, with densitometric quantification normalized to β-actin. The copy number of mitochondrial DNA relative to the GAPDH nuclear gene was determined using the quantitative real-time polymerase chain reaction (qRT-PCR). (**A**) PGC-1α: The top panel shows the mean relative expression normalized to β-actin, while the bottom panel displays representative Western blot images. (**B**) TFAM: The top panel presents the mean relative expression normalized to β-actin, and the bottom panel shows corresponding Western blot images. (**C**) COX-4: The top panel illustrates the mean relative expression normalized to β-actin, with the bottom panel showing representative Western blot images. (**D**) Copy number of mitochondrial DNA relative to the GAPDH nuclear gene. The results are expressed as the mean ± SD from at least three independent experiments. Statistical significance relative to the untreated control cells is indicated (* *p* < 0.05).

A statistically significant biphasic effect of NaHS on PGC-1α was shown by the bell-shaped curve of the protein levels elicited by the increasing supplementation of NaHS (Figure 3A). Dunnett’s post-test revealed a significant increase in protein levels in the cells treated with 1 × 10^−3^ M, 1.5 × 10^−3^ M and 3 × 10^−3^ M NaHS (*p* < 0.05). Additionally, the TFAM and COX-4 protein levels were significantly affected by the NaHS treatment (Figure 3B,C). Specifically, a statistically significant increase was observed in the cells supplemented with 1 × 10^−3^ M and 1.5 × 10^−3^ M NaHS for TFAM and in the cells supplemented with 1 × 10^−3^ M NaHS for COX-4 (*p* < 0.05). These findings indicate that the gasotransmitter’s stimulatory effect on the TFAM and COX-4 levels occurred at lower NaHS concentrations than its impact on the PGC-1α levels.

NaHS supplementation significantly impacted the relative copy number of mtDNA in the Caco-2 monolayer model. Treatment with NaHS in the range of 10^−7^–10^−4^ M increased the mtDNA copy number compared to the CTRL group, with notable differences observed at 5 × 10^−4^ M (*p* < 0.05). However, at higher concentrations of NaHS, a decline in the mtDNA copy number was detected. Taken as a whole, these results indicate a concentration-dependent biphasic effect of NaHS on mitochondrial biogenesis (Figure 3D).

### 3.4. Effects of Treatment with Different Concentrations of H_2_S-Releasing Donor on Antioxidant Defense and Inflammatory Response

**Figure 4:**
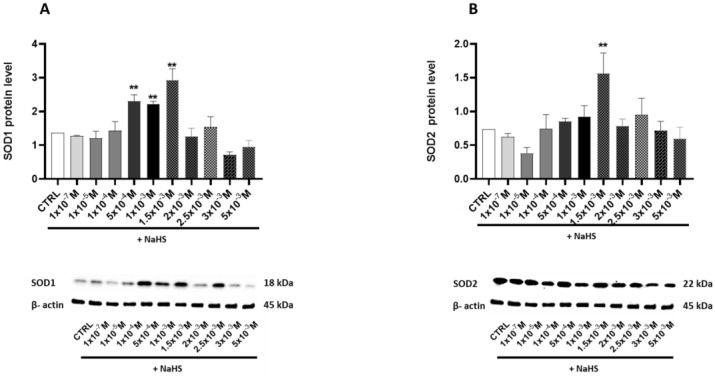
Relative expression levels of the key proteins involved in antioxidant defense in the Caco-2 monolayer model treated with increasing concentrations of NaHS. Protein abundance was assessed by Western blotting, with densitometric quantification normalized to β-actin. (**A**) SOD1: The top panel shows the mean relative expression normalized to β-actin, while the bottom panel displays representative Western blot images. (**B**) SOD2: The top panel presents the mean relative expression normalized to β-actin, and the bottom panel shows corresponding Western blot images. The results are expressed as the mean ± SD from at least three independent experiments. Statistical significance relative to the untreated control cells is indicated (** *p* < 0.01).

Protein levels of the antioxidant enzyme superoxide dismutase (SOD) were assessed to determine whether exposure to NaHS could activate cellular antioxidant defense mechanisms. The cytoplasmic (SOD1) (Figure 4A) and the mitochondrial (SOD2) proteins were evaluated (Figure 4B). Both enzymes exhibited a bell-shaped response to increasing NaHS concentrations. A significant increase was observed at 5 × 10^−4^ M, 1 × 10^−3^ M, and 1.5 × 10^−3^ M for the cytoplasmic SOD1, and at 1.5 × 10^−3^ M for the mitochondrial SOD2. At higher NaHS concentrations, the toxic effects of the gas became apparent, as indicated by a decline in the levels of both proteins.

**Figure 5:**
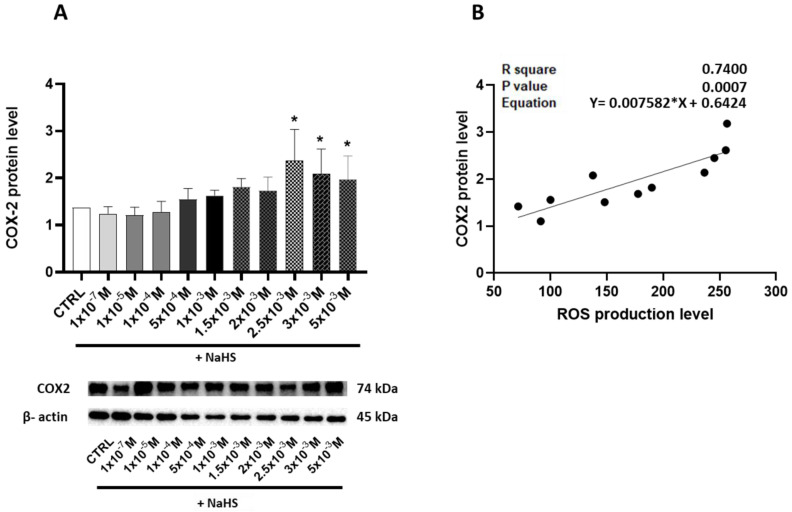
(**A**) Relative expression levels of COX-2, a key protein involved in the inflammatory response, in the Caco-2 monolayer model, treated with escalating concentrations of NaHS. Western blotting determined protein abundance, and densitometric quantification was normalized to β-actin. The top panel displays the mean relative expression of COX-2 normalized to β-actin, while the bottom panel shows representative Western blot images. (**B**) Linear regression showing the correlation between the relative expression of COX-2 and ROS production in the Caco-2 monolayer model treated with escalating concentrations of NaHS. Data are presented as the mean ± SD from at least three independent experiments. Statistical significance relative to the untreated control cells is indicated (* *p* < 0.05).

To explore the potential induction of an inflammatory state resulting from an oxidative imbalance, the pro-inflammatory enzyme cyclooxygenase-2 (COX-2) levels were measured. In the Caco-2 monolayer model treated with increasing concentrations of the H_2_S donor NaHS, a significant elevation in COX-2 levels was observed, particularly at higher NaHS concentrations, with the peak occurring at 2.5 × 10^−3^ M NaHS (Figure 5A). Moreover, the significant relationship between the COX-2 and ROS levels (R^2^ = 0.74, *p* = 0.0007) suggests a link between oxidative stress and inflammatory signaling (Figure 5B).

### 3.5. Effects of Treatment with Different Concentrations of H_2_S-Releasing Donor on Autophagy/Mitophagy and Apoptosis Response

**Figure 6:**
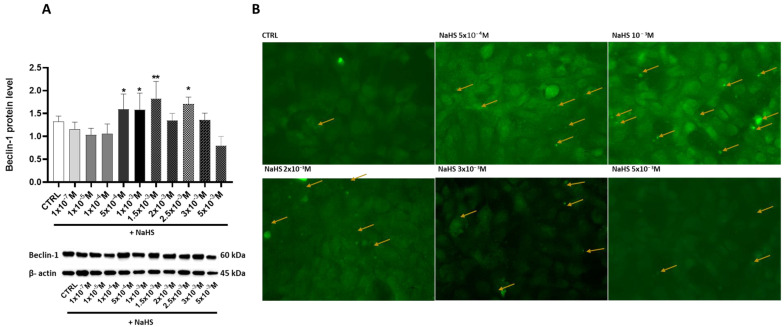
Protein levels of Beclin-1 and autophagosome formation in the Caco-2 monolayer model treated with increasing concentrations of NaHS. (**A**) Beclin-1 protein levels were analyzed by Western blot at varying concentrations of the H_2_S-releasing donor NaHS: the top panel shows the mean relative expression normalized to β-actin. In contrast, the bottom panel displays representative Western blot images. The results are expressed as the mean ± SEM from at least three independent experiments. Statistical significance relative to the untreated control cells is indicated (* *p* < 0.05, ** *p* < 0.01). (**B**) Representative 40× immunofluorescence (IF) images showing LC3 expression and autophagosome formation in the Caco-2 monolayer model treated with escalating concentrations of NaHS. The yellow arrows indicate autophagosomes.

This study investigated the effects of NaHS supplementation on cellular autophagy and mitophagy by analyzing the expression of Beclin-1, a key regulator of these processes, and monitoring autophagosome formation. Treatment with NaHS at various concentrations revealed significant increases in Beclin-1 expression at 5 × 10^−4^ M, 1 × 10^−3^ M, 1.5 × 10^−3^ M, and 2.5 × 10^−3^ M, with percentage increases of 20.21%, 19.53%, 40.5%, and 28.9%, respectively. In contrast, a remarkable 46.03% reduction in Beclin-1 expression occurred at 5 × 10^−4^ M (Figure 6A). The immunofluorescence data confirmed a correlation between Beclin-1 upregulation and autophagosome formation, emphasizing the role of NaHS in autophagy/mitophagy regulation (Figure 6B).

Building on these findings, this study further investigated the effects of NaHS on the apoptosis Caco-2 monolayer model, particularly at high concentrations. The Bax/Bcl-2 ratio was assessed to provide deeper insights into the mechanisms underlying apoptotic regulation.

**Figure 7:**
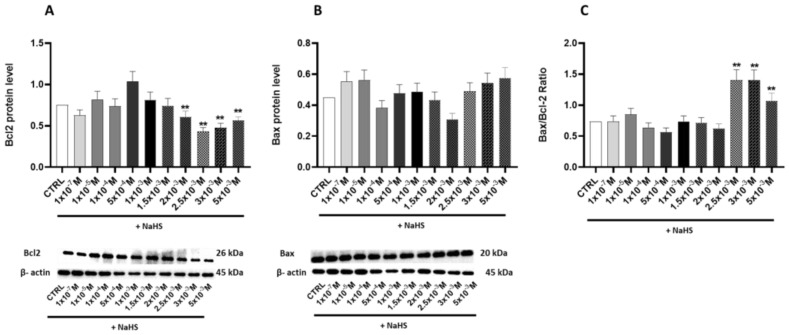
The relative expression levels of apoptosis proteins and the Bax/Bcl2 ratio. Western blot analysis was performed to quantify the expression of key apoptosis-related proteins, including Bax, Bcl2, and the Bax/Bcl2 ratio, in the Caco-2 monolayer model treated with escalating concentrations of NaHS. Protein abundance was assessed by Western blotting, with densitometric quantification normalized to β-actin. (**A**) Bcl2: The top panel shows the mean relative expression normalized to β-actin, while the bottom panel displays representative Western blot images. (**B**) Bax: The top panel presents the mean relative expression normalized to β-actin, and the bottom panel shows corresponding Western blot images. (**C**) Bax/Bcl2 ratio. Data are presented as the mean ± SD from at least three independent experiments. Statistical significance relative to the untreated control cells is indicated (** *p* < 0.01).

At concentrations of 2.5 × 10^−3^ M, 3 × 10^−3^ M, and 5 × 10^−3^ M, we observed a significant increase (*p* < 0.05) in this ratio, with gains of 82%, 76%, and 56%, respectively (Figure 7A–C). These results suggest that NaHS influences the mitochondrial apoptosis pathway, promoting mitochondria-dependent apoptosis and Bax translocation at high concentrations.

**Figure 8:**
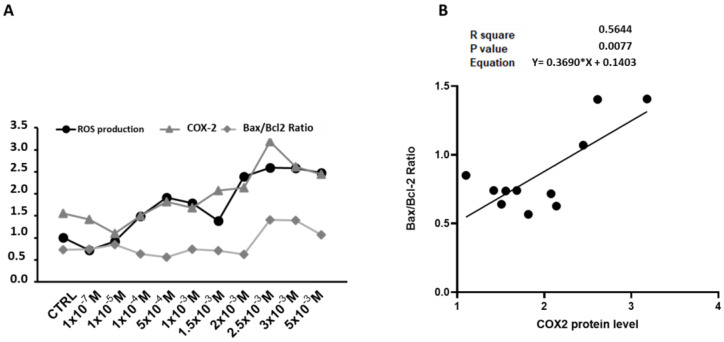
Relationship between ROS levels, COX-2 expression, and Bax/Bcl2 Ratio in the NaHS-treated Caco-2 monolayer model. (**A**) The expression trends of ROS levels, COX-2, and Bax/Bcl2. (**B**) Linear regression showing the correlation between the relative expression of COX-2 and the Bax/Bcl2 ratio in the Caco-2 monolayer model treated with escalating concentrations of NaHS. For each protein pair, the R^2^ value, *p*-value, and Pearson correlation equation are provided, indicating the strength and direction of the correlations.

The expression trends of ROS levels, COX-2, and the Bax/Bcl-2 ratio (Figure 8A) identify 1.5–2.0 × 10^−3^ M NaHS as a critical threshold. Below this concentration, ROS production, mitochondrial biogenesis, and autophagy/mitophagy exhibited a coordinated response. Above this threshold, the ROS levels increased alongside mitochondrial DNA damage, inflammation, and apoptosis. Correlation analyses between the Bax/Bcl-2 ratio and COX-2 expression revealed a significant positive correlation (R^2^ = 0.56, *p* = 0.008), indicating that an elevated COX-2 expression is associated with a shift toward apoptosis. These findings suggest that heightened ROS levels and reduced antioxidant capacity drive an inflammatory response that modulates the apoptotic pathways in NaHS-treated cells (Figure 8B).

## 4. Discussion

The present study aimed to investigate the balance between the protective and toxic effects of exogenous H_2_S in intestinal redox metabolism under non-stressed conditions, focusing on regulating mitochondrial biogenesis to maintain cellular homeostasis. We explored the therapeutic potential of NaHS, a physiological precursor of H_2_S, which impacts several mitochondrial functions, including the modulation of bioenergetics, antioxidant effects, and regulation of cell death [8]. Exogenous H_2_S donors have been utilized in various cellular and animal models, particularly for their impact on the cardiovascular and nervous systems [24]. In mammalian cells and tissues, H_2_S is typically present at low concentrations (1 × 10^−8^–3 × 10^−8^ M) [25], while the physiological levels in the human large intestine are in the millimolar range, with the free form in the micromolar range [26]. The literature suggests that micromolar concentrations of H_2_S can energize mitochondria in the gut by boosting cell respiration [27]. Therefore, a wide range of NaHS concentrations, from nano- to millimolar, was used in this study to determine appropriate therapeutic concentrations in the gut.

The data demonstrate that NaHS exhibits dose-dependent effects on Caco-2 cells, a monolayer model of intestinal cells. Treatment with the H_2_S precursor resulted in a biphasic curve of mitochondrial activity, with a slight increase at lower concentrations and significant impairment only at the highest millimolar concentrations. Cell viability was affected in a similar way, highlighting the nuanced role of H_2_S donors in intestinal cellular metabolism and suggesting potential thresholds for cytotoxic effects.

Since ROS produced during respiration by electron leakage along the mitochondrial ECT do not have exclusively deleterious roles but also provide redox signals for organelle maintenance [8], the production of these reactive species following treatment with increasing doses of NaHS was investigated. The results revealed a bell-shaped response, characterized by a non-significant increase in ROS levels at lower donor concentrations, followed by a pronounced and significant rise starting at 2 mM NaHS. This pattern indicates elevated ROS production and significant oxidative stress at higher concentrations.

The ROS imbalance at these concentrations was confirmed by the significant rise in oxidatively modified purines of mtDNA at 2 × 10^−3^ M NaHS compared to the untreated cells. Elevated levels of oxidants can stimulate crosstalk between the nucleus and mitochondria to regulate organelle biogenesis in severe sepsis [8]. Notably, H_2_S is among the molecules that induce mitochondrial biogenesis under non-stressed conditions [28,29].

In search of the molecular mechanism underlying the regulation of mitochondrial functions in Caco-2 cells by NaHS treatments, levels of the “master regulator” of mitochondrial biogenesis, PGC-1α, were investigated. Endogenous H_2_S has been found to trigger organelle biogenesis through the induction of PGC-1α [9]. Additionally, PGC-1α is persulfidated by endogenous molecules, positively influencing its nuclear localization and increasing its activity, leading to mitochondrial biogenesis [12]. Several reports show that exogenous H_2_S can upregulate the expression of PGC-1α in various tissues and cells, including the brain [30], liver [11], kidney [31], and heart [32,33], and increase its activity through persulfidation [11]. Mice administered with an H_2_S-releasing compound showed reduced cytosolic PGC-1α levels, increased nuclear levels of the protein, and increased expression of its downstream regulated genes [29]. Significant increases in PGC-1α appeared at slightly higher H_2_S donor concentrations than those of its downstream regulated TFAM protein and the mitochondrial mass marker COX-4. Based on these findings [11,30,31,32,33], it is suggested that exogenous H_2_S-related stimulation of mitochondrial biogenesis initially occurs through the activation of PGC-1α activity, followed by the upregulation of its expression at higher concentrations. A significant bell-shaped curve was also observed when the copy number of mtDNA was determined in the cells treated with increasing NaHS concentrations, further confirming the positive effects of moderate concentrations of the gasotransmitter on mitochondrial biogenesis. The drop in the mtDNA copy number and COX-4 protein levels at higher concentrations emphasizes the complexity of H_2_S biology, whose higher levels negatively affect mitochondrial bioenergetics [3].

The ability of cells to cope with increased ROS production was analyzed. Sulfide is an antioxidant that can scavenge one- or two-electron molecules, but low cellular concentrations limit its effectiveness [34]. H_2_S modulates the expression of antioxidant genes [15,16], so levels of the superoxide dismutase enzymes, cytoplasmic SOD1, and mitochondrial SOD2 were analyzed. NaHS administration promotes the expression of SOD enzymes in various tissues [35,36,37,38]. The NaHS dose-response biphasic curve for both enzymes indicates an early adaptive antioxidant response to support redox homeostasis, which appears to be overwhelmed at higher H_2_S donor concentrations, as shown by the drop in the two proteins at NaHS concentrations above 1.5 × 10^−3^ M. This concentration appears to represent a critical threshold beyond which exposure to NaHS disrupts the cellular redox balance in the gut.

The concerted response of both enzymes, not just the mitochondrial one, implies a ROS-induced response at the nuclear level, leading to increased antioxidant defense [39]. Since the imbalance between reactive species production and their elimination by protective mechanisms leads to inflammation [8], it was investigated whether H_2_S could mediate inflammation in Caco-2 cells under non-stressed conditions. Significant increases in the COX-2 inflammatory molecule were observed at millimolar NaHS concentrations when antioxidant defenses appeared to be overwhelmed, further supporting the suggested critical threshold. According to the newly defined role of the gasotransmitter as a driver of inflammation in several pathologies [40], results from this study may help develop novel approaches for gut inflammatory diseases.

The significant increase in ROS production and COX-2 expression at higher NaHS concentrations correlates with the activation of apoptotic pathways, as shown by the elevated Bax/Bcl-2 ratio. This suggests that oxidative stress and inflammation are key drivers of H_2_S-induced apoptosis at toxic concentrations.

Maintaining mitochondrial homeostasis by clearing damaged organelles is a crucial cellular mechanism. Impaired mitochondria can be removed through mitophagy, a specific form of autophagy responsible for proper mitochondrial turnover. Increasing attention has been paid to the role of autophagy/mitophagy in GI pathologies as a homeostatic mechanism to mitigate cell stress [41,42,43,44].

There has been debate on the roles of H_2_S in autophagy. This signaling molecule appears to be involved in both pro- and anti-autophagy pathways [45] and has also been demonstrated to regulate mitophagy [46]. Several reports highlight the exogenous H_2_S donor-mediated upregulation of mitophagy in various tissues [47,48]. To assess whether the cellular response to toxic levels of NaHS in the Caco-2 monolayer model involves autophagy or mitophagy as a defense mechanism against impaired mitochondrial metabolism, the levels of Beclin-1 and LC3 were evaluated. Beclin-1 plays a central role in autophagosome formation and, through its interaction with parkin [49], also contributes to the initiation of mitophagy [8]. LC3, a microtubule-associated protein, serves as a marker of autophagosome levels. Our results support an H_2_S-ROS crosstalk that affects autophagy/mitophagy as key compensatory mechanisms in response to H_2_S-induced mitochondrial stress. The upregulation of Beclin-1 and LC3 expression at intermediate H_2_S concentrations suggests an attempt to remove damaged mitochondria and restore cellular balance. However, these processes fail to mitigate mitochondrial damage at higher concentrations, leading to apoptotic cell death.

Data from this study confirm the role of H_2_S in inducing DNA damage/repair, thus affecting the cell cycle. Hydrogen sulfide gas has a cell growth regulatory role [50]. Indeed, the dual-faced behavior of H_2_S, cytoprotective or cytotoxic due to its concentration, is further evidenced by its ability to induce apoptosis at concentrations above a certain threshold [51]. Our results demonstrate that, above the threshold of 1.5–2 × 10^−3^ M, the molecule activates multiple cellular defense mechanisms, including antioxidant enzymes and autophagy/mitophagy pathways. These coordinated responses aim to counteract the molecular damage induced by the molecule itself and restore cellular homeostasis. However, when these defenses are overwhelmed, cells experience oxidative imbalance and inflammatory conditions, leading H_2_S to trigger apoptotic death in the compromised cells.

While our results may provide valuable insights, this study is limited by its reliance on an in vitro model. Results are mandatory for future studies aimed to validate them in vivo and explore the effects of exogenous H_2_S in more complex systems, such as organoids or animal models.

## 5. Conclusions

This study provides evidence for the biphasic effects of exogenous H_2_S in regulating mitochondrial function and biogenesis in intestinal epithelial cells under non-stress conditions. H_2_S enhances mitochondrial biogenesis at low-to-moderate concentrations, upregulates antioxidant defenses, and supports cellular homeostasis. However, at higher concentrations, H_2_S induces oxidative stress, disrupts mitochondrial function, and triggers inflammation. The activation of PGC-1α primarily mediates the cytoprotective effects of H_2_S, while excessive ROS production and COX-2 activation at higher concentrations lead to mitochondrial dysfunction and apoptosis. These findings highlight the therapeutic potential of H_2_S donors in GI diseases but emphasize the need for precise titration to avoid adverse effects.

## Figures and Tables

**Table 1:** Primer sequences.

Gene	Primers Foward	Primers Reverse
ND1	5′-TTCTAATCGCAATGGCATTCCT-3′	5′-AAGGGTTGTAGTAGCCCGTAG-3′
GAPDH	5′-CAGAACATCATCCCTGCCTCTAC-3′	5′-TTGAAGTCAGAGGAGACCACCTG-3′
D-loop	5′-CTGTTCTTTCATGGGGAAGC-3′	5′-AAAGTGCATACCGCCAAAAG-3′

ND1 GeneBank Accession number LC178901.1 Homo sapiens mitochondrial ND1 gene; GAPDH Gene Bank Accession number NG_007013.2 Homo sapiensglyceraldehyde-3-phosphate dehydrogenase (GAPDH), RefSeqGene on chromosome 12); D-loop GeneBank Accession number MZ647962.1, Homo Sapiens D-loop, complete sequence; mitochondrial.

## Data Availability

The original data presented in the study can be downloaded at: 10.6084/m9.figshare.28523570.

## Abbreviations

The following abbreviations are used in this manuscript:

3MST	3-Mercaptopyruvate Sulfurtransferase
ANOVA	Analysis of Variance
Bax	Bcl-2-associated X protein
BCL-2	B-cell lymphoma 2
CBS	Cystathionine β-Synthase
CO	Carbon Monoxide
COX-2	Cyclooxygenase-2
COX-4	Cytochrome c Oxidase Subunit 4
CTRL	Control
DCF	Dichlorofluorescein
DCFH-DA	2′,7′-Dichlorodihydrofluorescein Diacetate
DMEM	Dulbecco’s Modified Eagle Medium
DMSO	Dimethyl Sulfoxide
DNA	Deoxyribonucleic Acid
ETC	Electron Transport Chain
FBS	Fetal Bovine Serum
Fpg	Formamidopyrimidine DNA Glycosylase
GI	Gastrointestinal
H_2_S	Hydrogen Sulfide
HRP	Horseradish Peroxidase
IBD	Inflammatory Bowel Disease
IBS	Irritable Bowel Syndrome
IgG	Immunoglobulin G
LC3	Microtubule-Associated Protein 1A/1B-Light Chain 3
MEM	Minimum Essential Medium
MST	Mercaptopyruvate Sulfurtransferase
mtDNA	Mitochondrial DNA
MTT	3-(4,5-Dimethylthiazol-2-yl)-2,5-Diphenyl-Tetrazolium Bromide
NaHS	Sodium Hydrosulfide
NO	Nitric Oxide
OD	Optical Density
PBS	Phosphate-Buffered Saline
PBST	Phosphate-Buffered Saline with Tween-20
PGC-1α	Peroxisome Proliferator-Activated Receptor Gamma Coactivator 1-Alpha
qRT-PCR	Quantitative Real-Time Polymerase Chain Reaction
RIPA	Radioimmunoprecipitation Assay Buffer
ROS	Reactive Oxygen Species
RPM	Revolutions Per Minute
SDS-PAGE	Sodium Dodecyl Sulfate-Polyacrylamide Gel Electrophoresis
SEM	Standard Error of the Mean
SOD1	Superoxide Dismutase 1
SOD2	Superoxide Dismutase 2
SRB	Sulfate-Reducing Bacteria
TFAM	Mitochondrial Transcription Factor A
TBS	Tris-Buffered Saline
TBST	Tris-Buffered Saline with Tween-20
UV	Ultraviolet
